# Protein Stores Regulate When Reproductive Displays Begin in the Male Caribbean Fruit Fly

**DOI:** 10.3389/fphys.2020.00991

**Published:** 2020-08-06

**Authors:** Clancy A. Short, John D. Hatle, Daniel A. Hahn

**Affiliations:** ^1^Department of Entomology and Nematology, The University of Florida, Gainesville, FL, United States; ^2^Department of Biology, The University of North Florida, Jacksonville, FL, United States

**Keywords:** behavioral regulation, *lsp-*2, male courtship, nutrient storage, tephritidae, hexamerins, fat-body-derived signal, capital-breeding

## Abstract

Many animals exhibit reproductive behavior that requires expenditure of valuable nutrients. In males of many species, competitive energetically demanding displays and the development of sexual ornaments require prior accumulation of nutrient stores. Males must coordinate nutrient stores with ornament development and reproductive displays or they risk depleting their resources mid-development or mid-display, reducing their chance of mating. Males may use nutrient stores to regulate their reproductive behavior. Amino acid reserves may be important for reproduction, but the roles of amino acid stores in initiating maturation and reproductive behavior are less studied than fat stores. Insects store amino acids as hexamerin storage proteins. Many fly species use a specific hexamerin, larval serum protein 2 (LSP-2), as both a juvenile storage medium and to store protein consumed after adult eclosion. Protein stored as LSP-2 has previously been suggested to regulate reproduction in females, but no role has been proposed for LSP-2 in regulating male maturation. We use males of the Caribbean fruit fly, *Anastrepha suspensa*, a species with nutrient-intensive male sexual displays to test whether LSP-2 stores regulate male reproductive displays. We fed adult *A. suspensa* males a diet with or without protein, then assayed these males for *lsp-2* transcript abundance via qRT-PCR, LSP-2 protein abundance via Western blot, and reproductive display behavior via observation. We found that adult males with *ad libitum* dietary protein had greater *lsp-2* transcript and protein abundance, earlier sexual display behavior, and were more likely to exhibit sexual display behavior than protein-deprived adult males. We show that *lsp-2* knockdown via RNAi decreases the proportion of males exhibiting reproductive displays, particularly early in the onset of reproductive behavior. Our results suggest circulating LSP-2 protein stores regulate reproductive behavior in *A. suspensa* males, consistent with protein stores modulating reproduction in males with expensive reproductive strategies. Our results are consistent with hexamerin storage proteins performing dual roles of protein storage and protein signaling. Our work also has substantial practical applications because tephritid flies are a pest group and the timing and expression of male reproductive displays in this group are important for control efforts using the sterile insect technique.

## Introduction

Animals require nutrients for expensive life history transitions, especially reproductive maturation and engaging in nutrient-intensive reproductive behaviors (Houston et al., 2006; Harshman and Zera, 2007; Soulsbury, 2019). However, many animals live in nutrient-limited environments, and the availability of abundant nutrient sources may not match a time and place well-suited for reproductive behavior (e.g., Ubukata, 1984; Warner, 1987; Yuval and Bouskila, 1993; Soulsbury, 2019). Accordingly, animals have evolved strategies to mitigate this problem. Capital breeders solve this problem by storing nutrients when they are abundant, then using nutrient stores during maturation and reproduction (Houston et al., 2006; Soulsbury, 2019). Income breeders instead match their reproductive output to the immediate availability of nutrients in their habitat (Houston et al., 2006; Soulsbury, 2019). However, many animals rely on a combination of both income and capital to fuel their reproduction, so most reproductive strategies exist on the spectrum between fully capital or fully income breeding (Houston et al., 2006; Soulsbury, 2019). Thus, animals generally modulate their reproductive nutrient expenditure to avoid prematurely depleting their stores (Frisch, 1985; Yin et al., 1999; Gauthier-Clerc et al., 2001; Shelly and Kennelly, 2003; Arrese and Soulages, 2010; Smith and Spencer, 2012; Teal et al., 2013; Lebreton et al., 2017). Female mammals clearly regulate their reproductive behavior using their fat stores, as do female insects (Frisch’s fatness and fertility hypothesis; Frisch, 1985; Ellers, 1995; Glazier, 2000; Smith and Spencer, 2012; Badisco et al., 2013; Sieber and Spradling, 2015). Females are typically considered to have a higher cost of reproduction than males. However, reproduction is costly for males of many species because of the expensive nature of mating displays, as well as developing sexual ornaments and provisioning nuptial gifts (Simmons and Parker, 1989; Bleu et al., 2016). Males with low nutrient stores may pay a fitness cost because they miss breeding periods, are unable to perform competitive mating displays, and could die from starvation (Leather et al., 1983; Sandberg and Moore, 1996). Despite the costly nature of many male breeding strategies, the importance of nutrient reserves to male reproductive investment remains poorly investigated.

Many male reproductive displays require expensive signals (Soulsbury, 2019). Expensive signals can range from development of pre-breeding ornaments to pheromone-producing machinery to energetic fuel for behavioral displays. Amino acids are used to build sexual ornaments and the biochemical machinery needed for behavioral and chemical displays (e.g., proteins used to construct and maintain male ornaments or enzymes necessary to produce male pheromones). Storing amino acids prior to lekking or producing ornaments could be advantageous. In some birds and fiddler crabs, ornaments can interfere with foraging, so males may benefit from storing amino acids until they have acquired the resources needed to complete ornament growth (Moller et al., 1995; Allen and Levinton, 2007). Other species use lek mating systems where males aggregate at sites that are separated from resources and perform competitive reproductive displays to attract sexually selective females (Shelly, 2018). Many tephritid fruit flies use lek-mating systems and have amino acid intensive displays that are physically separated from amino acid sources, so these males may benefit from storing amino acids before traveling to lekking sites and beginning their competitive displays (Warburg and Yuval, 1997; Yuval et al., 1998; Benelli et al., 2014).

Males should delay or forego reproductive development in favor of additional foraging if they have insufficient amino acid stores to successfully reproduce. Although fat stores are associated with breeding behavior in some vertebrates (Pérez-Barberia et al., 1998; Wells, 2001; Welbergen, 2011), the importance of amino acid stores to reproductive displays remains largely uninvestigated. Vertebrates can undergo muscle histolysis when amino acid intake is insufficient to meet the needs of a life history transition like breeding (Brosnan, 2003; Parker et al., 2009), but vertebrates lack a dedicated store for amino acids, so understanding the role of protein stores in vertebrates can be challenging.

Insects can also undergo muscle histolysis to fuel male reproductive displays (Mitra et al., 2011), but insects, other hexapods, and some decapod crustaceans have a dedicated amino acid store, hexamerin storage proteins (Burmester, 1999; Terwilliger et al., 1999; Capurro et al., 2000; Wheeler et al., 2000; Tawfik et al., 2006; Tokar et al., 2014; Xie and Luan, 2014). Hexamerin storage proteins are evolutionary derived from crustacean hemocyanin respiratory proteins that have lost their copper binding sites for oxygen (Burmester, 1999; Terwilliger et al., 1999). Hexamerin storage proteins, often abbreviated to “hexamerins,” are abundant blood proteins that circulate as hexamers consisting of ∼70 kDa subunits (Burmester, 1999). Hexamerins are produced by the fat body and secreted into the hemolymph in both juveniles and adults of all insects thus far studied, but in holometabolous larvae they are reabsorbed by the fat body shortly before metamorphosis (Burmester, 1999). Hexamerin accumulation is associated with providing anabolic substrates for molting and metamorphosis in both sexes, as well as female reproduction (Pan and Telfer, 1996; Wheeler and Buck, 1996; Burmester, 1999; Capurro et al., 2000; Wheeler et al., 2000; Hahn et al., 2008; Arrese and Soulages, 2010). Quantifying and manipulating hexamerin levels could disentangle the effects of current dietary protein availability from the effects of protein storage to explicitly test the extent to which protein storage regulates maturation and reproductive behavior. RNAi knockdown of hexamerins in females of the bean bug, *Riptortus pedestris*, delays the nymphal-adult molt and decreases the number of eggs a female lays (Lee et al., 2017). These phenotypes are also induced by starvation (Kim and Lim, 2014; Rahman et al., 2018), suggesting that hexamerins may coordinate nutrition with life history transitions in some female insects. However, the extent to which hexamerins regulate reproductive behavior in males was previously untested.

Tephritid fruit flies can be used to explore the relationships between protein stores and male reproduction because males of many tephritid species show a clear relationship between protein availability and male behavioral maturation (Teal et al., 2013). Adult tephritids may ingest small amounts of amino acids by feeding on bacteria and the residual nutrients that are found on fruit, bird droppings, and the surfaces of leaves (Aluja et al., 1999). These resources vary in availability and amino acid content, suggesting many tephritids experience amino acid limitation. Adult males of many tephritid species perform complex, intensive mating displays including participation in leks. Lekking sites are spatially separated from amino acid sources and feeding for teprhitid fruit flies (Benelli et al., 2014). Male tephritids need amino acids to mature, form ejaculate, build the molecular machinery to synthesize pheromones, and maintain the musculature necessary for producing complex courtship songs (Marchini et al., 2003). Dietary protein availability can accelerate the onset and increase the frequency of lekking behavior in males of many tephritid species (Warburg and Yuval, 1997; Teal et al., 2013; Collins et al., 2014), i.e., protein deprivation delays reproductive behavior. Lekking behavior itself also seems to expend amino acids; males begin lekking with high concentrations of total body soluble protein and end lekking behavior with low concentrations of soluble protein (Warburg and Yuval, 1997; Yuval et al., 1998). The depletion of whole-body soluble protein suggests that lekking male tephritids rely on amino acid capital accumulated before they enter leks, but the storage mechanisms for amino acid capital are uninvestigated.

One candidate hexamerin that may provide the stored amino acids necessary for male mating in tephritid flies is larval serum protein 2 (LSP-2). LSP-2 was first identified in the vinegar fly *D. melanogaster* (Roberts et al., 1977), where it is produced in the fat body and secreted into the hemolymph during larval and adult life (Beneš et al., 1990). Larval LSP-2 is then reabsorbed by the fat body and epidermal cells shortly before metamorphosis, presumably to provide anabolic substrate for metamorphosis and cuticle formation (Lepesant et al., 1978; Beneš et al., 1990; Tsakas et al., 1991; Burmester, 1999; but see Chrysanthis et al., 1994). LSP-2 appears to be the hexamerin responsible for storing amino acids consumed during the adult stage in females of higher fly (Suborder: Brachycera) species (Beneš et al., 1990; Chrysanthis et al., 1994; Capurro et al., 2000; Hahn et al., 2008; but see Burmester et al., 1998). LSP-2 is accumulated with adult protein feeding and depleted with egg production in the vinegar fly *Drosophila melanogaster*, the housefly *Musca domestica*, and the flesh fly *Sarcophaga crassipalpis* (Beneš et al., 1990; Chrysanthis et al., 1994; Capurro et al., 2000; Hahn et al., 2008; but see Burmester et al., 1998). We hypothesize that the hexamerin LSP-2 stores amino acids prior to lekking behavior in tephritid fruit fly males, and that males regulate their lekking behavior based on their LSP-2 stores. If LSP-2 acts as a protein store in tephritid fruit fly males, then *lsp-2* transcript abundance should increase in response to protein feeding, and LSP-2 protein should accumulate during continued protein feeding. We predict that *lsp-2* knockdown should suppress male reproductive behavior.

The Caribbean fruit fly *Anastrepha suspensa* Loew is a competitive lekking tephritid species (Burk, 1983). *Anastrepha suspensa* is a pest of guava, peach, Surinam cherry, tropical almond, and loquat, and has a host range of more than 90 fruits (Baranowski et al., 1993). Like many other tephritids, *A. suspensa* may feed on bacteria, fungi, and animal feces (Aluja et al., 1999), but variation in the availability and amino acid content of these food sources, and predation risks associated with foraging (Burk, 1983), may limit amino acid intake. For reproduction, males form leks where groups of males disperse themselves across individual leaf territories within one region of a plant and the males compete for a limited number of choosy females with wing fanning, song, and pheromone displays (Burk, 1983). Males that do not join leks can attempt to intercept females while they are ovipositing at fruit, but these non-lekking males have a much lower chance of mating success than lekking males (Burk, 1983). Protein in the adult diet of *A. suspensa* increases lek initiation and participation behavior, calling behavior, and mating success (Teal et al., 2013). However, the relationship between adult protein feeding and the age when calling and lekking behavior begin has not been investigated in this fly species. Here we show that providing protein in the adult diet of males of the tephritid fruit fly *Anastrepha suspensa* increases their *lsp-2* transcript and LSP-2 protein abundances. Dietary protein also causes both earlier sexual displays and causes a greater proportion of males to exhibit sexual display behavior. Knocking down *lsp-2* transcript abundance using RNAi reduces the proportion of males exhibiting sexual display behavior despite dietary protein availability, mimicking the protein-deprived courtship phenotype. Taken together, our results demonstrate that *A. suspensa* males can use capital protein stores, in the form of LSP-2, to regulate the timing of behavioral reproductive maturation and the probability that a male will engage in reproductive behavior, the first report of hexamerins regulating male reproduction. In addition to building basic understanding of the regulation of insect reproduction, our results have practical application. Because *A. suspensa* is a model for sterile male release programs to control pest tephritid populations, and sterile insect technique is predicated on males exhibiting appropriate lekking behavior, understanding the relationship between LSP-2 and accelerated male mating behavior could contribute to greater efficacy and cost efficiency of sterile male programs.

## Materials and Methods

### Fly Sampling and Sexual Display Analysis

**FIGURE 1:**
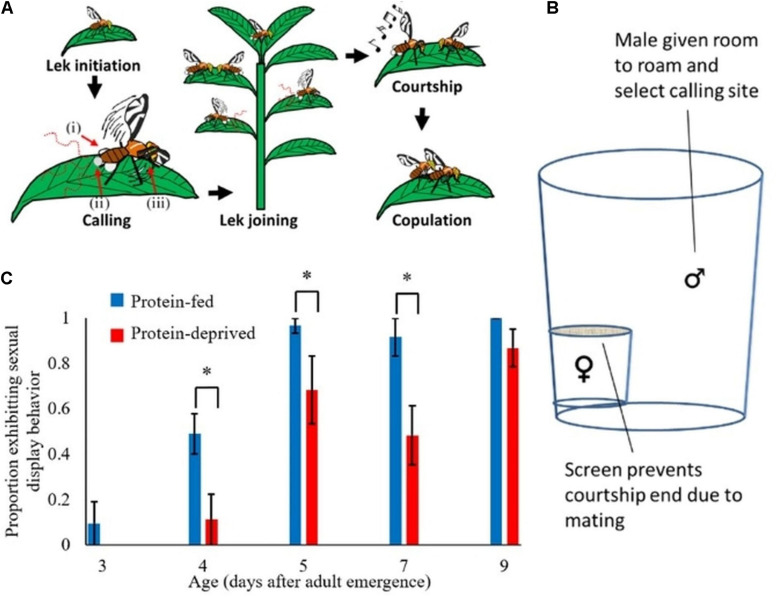
The complicated sexual display behavior of *Anastrepha suspensa* is sensitive to protein deprivation. **(A)** *Anastrepha suspensa* males follow a stereotyped sequence of behaviors leading to mating. Briefly, a male will select a site and defend it against other males (lek initiation), then evert his pleural and anal glands to release pheromone (calling behavior), then other males will select adjacent sites and begin their own calling behavior (lek joining). A female will come to the lek and males will begin courtship song and dance (courtship), and finally a female may allow copulation (Burk, 1983; Benelli et al., 2014). For our experiments, we measured the proportion of males exhibiting calling behavior, and refer to calling throughout as sexual display behavior. Calling behavior is comprized of 3 events: (i) the eversion of the pleural and (ii) anal glands to release pheromones, and (iii) the fanning of wings that disperses their pheromones (Nation, 1972; Benelli et al., 2014). **(B)** The assay design for inducing sexual display behavior included one male and one female in each container, and 20 containers were run in parallel. Though calling normally occurs within a lek, it can also occur in isolation, and our males were isolated from other males to reduce confounding factors. Only males that displayed at least 2 of the 3 calling behaviors were scored as exhibiting sexual display behavior. **(C)** Protein-fed males began sexual displays earlier than protein-deprived males, and protein-fed males called significantly more than protein-deprived males 4, 5, and 7 days after adult eclosion (Pearson’s Chi-squared, *indicates *p* < 0.05 (*χ*^2^ > 5.9), *p* > 0.25 (*χ*^2^ < 1.3) for all nonsignificant comparisons). In a reduced model explaining sexual display behavior, age and its interaction with diet were both significant (Table 1; *n* = 251, *n* = 20–28 for each diet × age combination). Whiskers represent standard error.

For our experiments we used a colony of *Anastrepha suspensa* (Loew 1862) (Diptera: Tephritidae) that originated from South Florida, United States in the summer of 1998 (Handler and Harrell, 2001). Our maintenance procedures included *ad libitum* access to larval and adult diets, as described by Teets et al. (2019); species background in Aluja et al. (1999). To test the effects of dietary protein on *lsp-2* transcript abundance, LSP-2 protein abundance, and behavioral reproductive maturation, we used two contrasting experimental diets; a protein-containing diet (3:1 sucrose: enzymatic hydrolyzed brewers yeast from MP Biomedical, Solon, OH, United States) or a protein-deficient diet (sucrose only). Freshly eclosed adult males were caged in groups of ten and given *ad libitum* access to water and only one of the two experimental diets. Males were sampled at adult eclosion, and 1, 2, 3, 4, 5, 7, and 9 days after adult eclosion. Because females were not caged with males, all males assayed were virgin and naïve to females. For sampling, males aged 3–9 days after adult eclosion from both protein-rich and protein-deficient diets were assayed for stereotyped sexual display behavior. In *A. suspensa*, full reproductive behavior includes lek initiation, calling behavior, lek joining, courtship and copulation (Burk, 1983; described in Figure 1A). In our study we focus on calling behavior, comprized of (i) the eversion of the pleural and (ii) anal glands to release pheromones, and (iii) the fanning of wings that disperses their pheromones (Aluja et al., 1999; Benelli et al., 2014). Briefly, males were placed in individual containers and provided with female olfactory and visual cues from 10 to 14 day post-eclosion females that were previously fed protein *ad libitum*, and thus were fully reproductively mature. Calling assays were run from 15:00 to 17:00, coincident with peak courtship timing (Burk, 1983; Landolt and Sivinski, 1992). Each assay began by placing males into the arena (plastic deli cup, 0.95 L, 105 mm diameter) and giving males a 10-minute acclimation period before females were added to the smaller screened-off container (plastic deli cup, 35 mL, 40 mm diameter) within the arena (set-up shown in Figure 1B). After an additional 10-minutes of acclimation, the flies were monitored for whether they exhibited the calling behaviors described above. Only males that exhibited at least two of the three calling behaviors described above were scored as exhibiting sexual displays. Behavioral assays were conducted at 23°C and at 30–50% humidity (our standard laboratory conditions). Because each male was frozen for subsequent biochemical analysis after being behaviorally assayed, each male was tested for sexual display behavior only once. Ten flies of the same age reared on protein-containing and protein-deficient diets were placed in separate arenas and were observed simultaneously. Each observation session used only one cohort of flies, and included both protein-fed and protein-deprived males, preventing cohort to cohort variation from being falsely attributed to age or diet. Although we did not directly measure feeding, we did measure total soluble protein content of whole male bodies. If males that had access to dietary protein were indeed feeding, we predict these males should have had higher total body soluble protein than their protein-deprived counterparts. To test whether our design had generated changes in the total soluble protein content of whole male bodies, we used BCA assays (Pierce^TM^ BCA kit, Thermo Fisher Scientific, Waltham, MA, United States) to measure total soluble protein content of whole male bodies.

### Characterization of LSP-2

Because the genome and proteome of *A. suspensa* remain unpublished, the sequence of LSP-2 in *A. suspensa* is still unpublished, so our study characterized LSP-2 protein from larval and adult blood. To characterize LSP-2 in *A. suspensa*, blood was drawn from 5 wandering 3rd instar larvae, 15 freshly eclosed adult males and 15 freshly eclosed adult females, as well as 15 protein-fed males and 15 protein-fed females 8 days after adult eclosion. Blood proteins were separated by loading 2 to 5 μg of protein onto a 10% Mini-PROTEAN^®^ TGX^TM^ Precast PAGE Gels (Bio-Rad, Hercules, CA, United States) with Laemmli Sample Buffer (Bio-Rad, Hercules, CA, United States). SDS-PAGE was run at 145 V for 75 minutes in a Mini-PROTEAN^®^ II Electrophoresis Cell (Bio-Rad, Hercules, CA, United States) according to the manufacturer’s instructions. To visualize bands, gels were stained with Coomassie Biosafe (Bio-Rad, Hercules, CA, United States). One band (∼72 kDa) was highly abundant in wandering 3rd instar larvae and both sexes 8 days after adult eclosion, as predicted for LSP-2 (Supplementary Figure 1A). The band was excised from the lane loaded with blood of protein-fed males 8 days after adult eclosion and LC-MS/MS for peptide identification was performed at the UF ICBR Proteomics core facility. LC-MS/MS of the band detected 9 short peptides that matched the predicted LSP-2 sequence of *C. capitata* (Supplementary Figure 1B; Sequence ID: XP_004530681.1), confirming that LSP-2 circulates in the blood of larval and adult *A. suspensa* of both sexes.

### Quantification of *lsp-2* Transcript Abundance

To generate cDNA, samples were homogenized and RNA pellets were extracted using TRIreagent^®^ (Invitrogen, Carlsbad, CA, United States) according to the manufacturer’s instructions, except 1-bromo-3-chloropropane (Sigma-Aldrich, St. Louis, MO, United States) was used instead of chloroform for phase separation. RNA quality was checked by selecting every 24th RNA extraction and using 5 μg of RNA for bleach gel electrophoresis (Supplementary Figure 2; Aranda et al., 2012). To generate cDNA from RNA, the SuperScript^®^ SSOAdvanced First-Strand Synthesis Reagents kit (Invitrogen, Carlsbad, CA, United States) was used according to the manufacturer’s instructions using 1 μg of total RNA per 20 μl reaction and oligo (dT)20 as the reverse transcription primer.

We used degenerate primers (IDT, Coralville, IA, United States) to isolate a 750 bp fragment of the *lsp-2* mRNA transcript from wandering 3rd instar *A. suspensa* larvae (Supplementary Table 1). Degenerate primers were developed from the consensus region of *lsp-2* in the melon fly, *Bactrocera cucurbitae*, snowberry maggot, *Rhagoletis zephyria*, and *C. capitata* (GenBank sequences: XM_011186009.1, XM_017622567.1, XM_004530624.). Sanger sequencing (performed by GeneWiz, South Plainfield, NJ, United States) revealed our 750 bp fragment had high similarity with *lsp-2* from other flies, so likely represents a partial sequence of the *A. suspensa lsp-2* mRNA (Supplementary Table 2). From this sequence, we developed qRT-PCR primers for *lsp-2* (PrimerQuest^®^ design tool by IDT, Coralville, IA, United States; Supplementary Table 1). Primers described by Nakamura et al. (2016) for the housekeeping gene *rp18* in the West Indian fruit fly, *Anastrepha obliqua* (Supplementary Table 1) were used to estimate the transcript abundance of *rp18* as an *A. suspensa* reference gene. qRT-PCR was run with an annealing temperature of 54°C using SsoAdvanced^TM^ Universal SYBR^®^ Green Supermix (Bio-Rad, Hercules, CA, United States) according to manufacturer’s instructions and the CFX Connect Real-Time PCR Detection System (Bio-Rad, Hercules, CA, United States). Primer specificity was verified by melt curve, gel electrophoresis, and Sanger sequencing of the subsequent single-band PCR product (performed by GeneWiz, South Plainfield, NJ, United States).

To locate *lsp-2* transcripts in adult males, *lsp-2* transcript abundance was examined in the head, legs, and abdomen of protein-fed and protein-deprived males 4 days after adult eclosion (RNA extracted and cDNA synthesized as described above). Heads, legs, and abdomens were pooled in groups of tissues from five individual flies (30 legs/pool), each pool was replicated four times (*n* = 12). *lsp-2* transcripts were clearly present in both the head and abdomen (Supplementary Figure 3), confirming that using entire carcasses for RNA and protein extraction was appropriate for estimating *lsp-2* transcript abundance between protein-fed and protein-deprived males. Whole bodies were used to estimate *lsp-2* transcript abundance across all ages and dietary treatments.

To test for effects of dietary protein on *lsp-2* transcript abundance, we ran qRT-PCR on samples described above that were collected during the first 3 days of adult life as well as males up to 9 days after adult eclosion that were phenotyped for sexual display behavior. Samples were randomized across qRT-PCR plates and run alongside 3 concentrations of internal standard comprized of mixed cDNA from randomly selected samples. C_*q*_ values were calculated using CFX Manager^TM^ Software’s (Bio-Rad, Hercules, CA, United States). *lsp-2* C_*q*_ was divided by the housekeeping gene *rp18* C_*q*_ to calculate relative *lsp-2* transcript abundance using the 2^–ΔΔ^*^*CT*^* method (Livak and Schmittgen, 2001). *rp18* transcript abundance was not significantly influenced by age, diet, or RNAi treatment in any experiment (LMM, starting with the model 2^–^*^*rp18Ct*^* ∼ Diet ^∗^ Age, including cohort as a random factor, and reducing via backward step AIC, *p* > 0.244 for any model, full or reduced, *n* = 207).

### Quantification of LSP-2 Protein Abundance

LSP-2 protein abundance was estimated with western blots. Protein pellets were extracted from the same TRIreagent^®^ (Invitrogen, Carlsbad, CA, United States) homogenate as the RNA, according to manufacturer’s instruction. The entire fly body was used in this homogenate. Protein pellets were dissolved in lysis buffer (Kopec et al., 2017). To perform western blots, an anti-LSP-2 antibody for *A. suspensa* was developed (LifeTein, Somerset, NJ, United States). The polyclonal primary rabbit antibody reacted to the epitope sequence C-NFIHGEHKDDMEAVNQLGN translated *in silico* from our *A. suspensa lsp-2* fragment. To prepare for western blotting, protein concentration in extracts was measured by Pierce^TM^ BCA assay (Thermo Fisher Scientific, Waltham, MA, United States). Proteins were separated using the SDS-PAGE procedure described above and 2.5 μg of total protein. Proteins were transferred from gels to polyvinylidene fluoride membranes (Bio-Rad, Hercules, CA, United States) using a *Trans-*Blot^®^ Turbo^TM^ Transfer System (Bio-Rad, Hercules, CA, United States) according to the manufacturer’s instructions. Immune probing was conducted using an anti-LSP-2 antibody concentration of 190 μg/L, and a mouse monoclonal Anti-α-Tubulin antibody (used as a loading control, produced by Sigma-Aldrich, Carlsbad, CA, United States) concentration of 200 μl/L for primary incubation. Secondary incubation used anti-rabbit and anti-mouse IgG HRP-conjugated goat antibody at a concentration of 100 μl/L (EMD Millipore Corp, Burlington, MA, United States). Bands were visualized with Clarity Max^TM^ Western ECL Blotting Substrates (Bio-Rad, Hercules, CA, United States) and chemiluminescence was detected with a ChemiDoc^TM^ MP Imaging System (Bio-Rad, Hercules, CA, United States). To account for technical differences between gels and membranes, samples were randomized and an internal protein standard solution (made by mixing protein from randomly selected samples) was included on every blot. LSP-2 intensity was divided by α-Tubulin intensity to calculate normalized LSP-2 protein abundance. We expected that tubulin protein abundance would be stable through time, but tubulin protein abundance was significantly influenced by the interaction of diet and age, with tubulin protein content increasing with age in protein-fed males, and decreasing with age in protein-deprived males (LMM, square root (Standardized tubulin fluorescence) ∼ Diet ^∗^ Age, including cohort as a random factor, Diet^∗^Age had an effect size of 153, S.E. of 52.9, *t* = 2.89, *p* < 0.01, Diet had an effect size of 511, S.E. of 273, *t* = 1.87, *p* = 0.06, Age had an effect size of 67.3, S.E. of 37.3, *t* = 1.81, *p* < 0.07, *n* = 207). The diverging tubulin concentration with age could affect the interpretation of our results. However, when the difference in tubulin levels between protein-fed and protein-deprived males was largest and had the lowest *p*-values (7 and 9 days after adult eclosion), protein-fed males had greater tubulin levels than protein-deprived males. The higher levels of tubulin in protein-fed males compared to protein-deprived males biases our results toward not finding a difference in LSP-2 protein content between protein-fed and protein-deprived males. Thus, our detection of higher LSP-2 abundance in protein-fed males than in protein-deprived males should be considered a conservative interpretation.

### RNAi Knockdown of *lsp-2*

To disentangle the effects of dietary protein availability from protein storage, we experimentally knocked down *lsp-2* transcript abundance using RNAi. Adult flies, 12–24 h after eclosion, were immobilized on ice, then injected with 0.6 μg of either *lsp-2* dsRNA or *gfp* dsRNA (a control treatment) in elution buffer (Thermo Fisher Scientific, Waltham, MA, United States). To create the dsRNA, we used the MEGAscript^TM^ RNAi Kit (Thermo Fisher Scientific, Waltham, MA, United States), according to the manufacturer’s instructions. We used the primers listed in Supplementary Table 1 (IDT, Coralville, IA, United States), and used our internal cDNA standard (described above) and a GFP plasmid (pGLO^TM^ Plasmid, Bio-Rad, Hercules, CA, United States) as templates for synthesis of *lsp-2* and *gfp* amplicons, respectively, (sequences in Supplementary Table 3). The amplicons were then transcribed into dsRNA overnight. To test for an effect of RNAi treatment on male sexual display behavior, males were caged in groups of ten and given *ad libitum* access to water and a protein-containing diet or protein-deficient diet (described above). Males were assayed for sexual display behavior as described above at 4 and 7 days after adult eclosion, then preserved for analysis of *lsp-2* transcript abundance to determine RNAi efficacy using the qRT-PCR methods described above. To verify that sexual display behavior differences between dsRNA treated flies were not due to off target differences in dietary protein feeding behavior (i.e., to show that *lsp-2* RNAi male flies were not protein-starved), we estimated total body soluble protein content in male flies using BCA kits (Thermo Fisher Scientific, Waltham, MA, United States). We verified that protein-fed anti-*lsp-2* dsRNA injected flies did not have detectably lower total protein content than protein-fed anti-*gfp* dsRNA injected flies (LMM, Total protein ∼ dsRNA treatment, cohort as random factor, RNAi treatment had an effect size of 0.002, S.E. of 0.0498, *p* = 0.97, *n* = 27).

### Statistical Analyses

To test whether differences in sexual display behavior, *lsp-2* transcript abundance, and LSP-2 protein abundance were different between treatment groups, we used combinations of linear mixed models (LMM) and generalized linear mixed models (GLMM). Models are listed in Table 1 and all include cohort as a random factor. Males from the day of eclosion were not included in any of our models because these males did not consume either diet. All models began as rich models with interactions and were reduced using backward step AIC, removing the term with the highest *p*-value. Once no more terms could be removed without raising the AIC less than 2, the final reduced model comprised the remaining terms. Only reduced models are shown in Table 1, except for the fully parameterized model explaining sexual display behavior using age, diet, *lsp-2* transcript, and LSP-2 protein abundance. For RNAi experiments, males 4 and 7 days after adult eclosion were analyzed separately because protein-deprived males exhibited no sexual display behavior 4 days after adult eclosion, preventing the use of a single generalized linear model. Males 4 days after adult eclosion were analyzed with a Chi-square test, while males 7 days after eclosion were analyzed with a mixed generalized linear model. All analysis of our data was run in the R (3.5.1) statistical program (R Core Team, 2018), using the packages *lme4*, *ggplot2*, and *mosaicData*. We also used Chi-squared and two sample *T*-tests corrected with false discovery rate corrections as post-hoc linear contrasts for models. Chi-squared tests were used for post-hoc analysis of mixed generalized linear models, while two sample *T*-tests were used for post-hoc analysis of linear models. For a more detailed description of our statistical tests, our code has been made publicly available in the Supplementary Files.

**TABLE 1:** Results of statistical linear models.

Model	Variable	Estimated effect size	Std. error	*z* or *t* value	Pr(> |*z*|)	Sig
**(A)** Generalized Linear Mixed Model (GLMM):	Intercept	4.10	0.768	–5.34	9.49E-08	***
Behavior ∼ Age * Diet (df = 246)	Age	0.675	0.120	5.62	1.94E-08	***
	Diet	2.40	1.61	–1.49	0.136	
	Age*Diet	0.960	0.367	2.62	0.00891	**
**(B)** Linear Mixed Model (LMM): *lsp-2* transcript	Intercept	0.6112	0.317	1.93	0.0570	
abundance ∼ Age * Diet (df > 75)	Age	0.0784	0.0613	–1.28	0.202	
	Diet	1.37	0.448	3.06	0.0024	**
	Age * Diet	0.242	0.0703	2.79	0.00582	**
**(C)** LMM: LSP-2 protein abundance ∼ Age +	Intercept	2.92	0.419	6.97	5.01e-06	***
*lsp-2* transcript abundance (df > 14) (Diet	Age	–0.383	0.0710	–5.39	1.95e-07	***
absent from model)	*lsp-2* transcript abundance	0.454	0.0903	5.03	1.07e-06	***
**(D)** Rich Model: GLMM: Sexual Display	Intercept	4.79	0.885	–5.42	6.03E-08	***
Behavior ∼ Age * Diet + LSP-2 protein + *lsp-2*	Age	0.0801	0.150	5.32	1.01E-07	***
transcript (df = 200)	Diet	2.54	1.84	–1.38	0.167	
	Age*Diet	0.893	0.413	2.16	0.031	*
	LSP-2 protein abundance	–0.0340	0.124	–0.0276	0.783	
	*lsp-2* transcript abundance	0.207	0.154	1.347	0.178	
**(E)** Reduced Model: GLMM: Sexual Display	Intercept	5.43	0.777	–6.98	2.94E-12	***
Behavior ∼ Age + (Age × Diet) (df = 203)	Age	0.910	0.135	6.74	1.61E-11	***
	Age × Diet	0.466	0.113	4.13	3.61E-05	***
**(F)** *lsp-2* transcript abundance ∼ Age + Diet +	Intercept	4.40	0.898	4.90	0.00023	***
dsRNA treatment (df > 9)	Age	–1.76	0.863	–2.04	0.0721	
	Diet	3.08	0.833	3.70	0.000339	***
	dsRNA treat.	2.30	0.834	–2.75	0.00687	**
**(G)** GLMM: Day 7	Intercept	1.40	0.455	–3.01	0.00202	**
behavior ∼ Diet (dsRNA treat. Absent from model) (df = 67)	Diet	3.00	0.646	4.64	6.42e-06	***

The response variable precedes “∼,” while explanatory variables follow “∼.” All models include cohort as a random factor. Age is a numeric factor. “*” indicates *p* < 0.05, “**” indicates *p* < 0.01, “***” indicates *p* < 0.001.

## Results

### Protein-Fed Males Exhibit Sexual Display Behavior Earlier and More Often

To test for effects of dietary protein on behavioral reproductive maturation and sexual display behavior, we sampled protein-fed and protein-deprived flies over the course of their reproductive maturation and assayed for stereotyped sexual display behavior (Figure 1A), *lsp-2* transcript abundance, and LSP-2 protein abundance. Males fed the experimental diet containing protein had significantly higher total soluble protein than males fed the sucrose-only diet, demonstrating substantial protein feeding (LMM, Total protein ∼ Diet, cohort as random factor, Diet had an effect size of 1.06, S.E. of 0.431, *p* = 0.014, *n* = 207; Supplementary Figure 4). Protein-fed males began sexual display behavior 1 day earlier in adult life and a greater proportion exhibited sexual display behavior 4–7 days after adult eclosion compared to protein-deprived males (GLMM, Model A, Age^∗^Diet had an effect size of 2.40, S.E. of 1.61, *p* < 0.01, *n* = 251; in Chi-square post-hoc analysis for significant comparisons *p* < 0.05, *χ*^2^ > 5.9, for all non-significant comparisons *p* > 0.25, *χ*^2^ < 1.3, *n* = 20–28 for each diet × age combination; Figure 1C); although male sexual display behavior increased with age in both diet groups (GLMM, Model A, Age had an effect size of 0.675, S.E. of 0.120, *p* < 0.001, *n* = 251; Figure 1C). However, our initial experiment did not disentangle the effects of dietary protein availability from the effects of protein stores on the initiation of mating behavior.

### Protein-Fed Males Have Higher *lsp-2* Transcript and LSP-2 Protein Abundance

**FIGURE 2:**
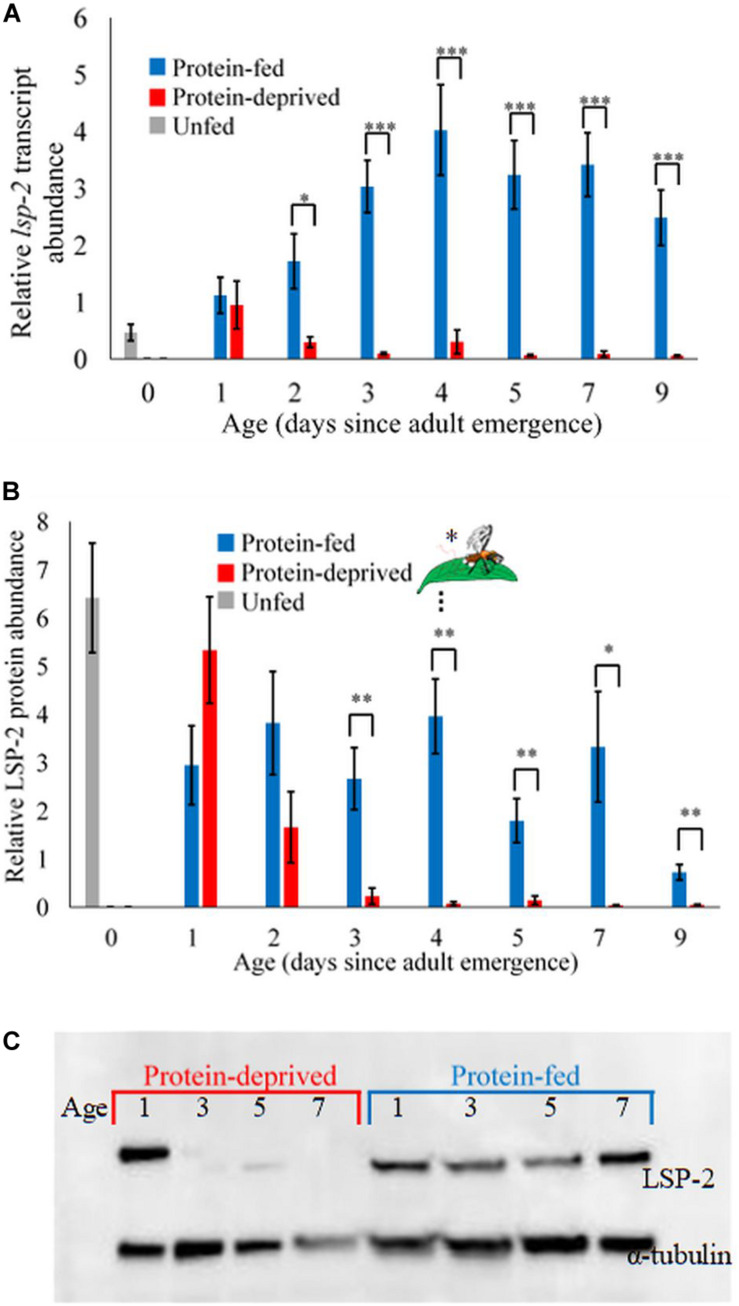
*lsp-2* transcript and protein abundance in whole animals were sensitive to protein feeding. **(A)** *lsp-2* transcript abundance increased with age in protein-fed males, but was almost undetectable in protein-deprived males 2 through 9 days after adult eclosion (Two sample *T*-test, *indicates *p* < 0.05 (*t* > 2.85), ***indicates *p* < 0.001 (*t* > 4.5), *p* > 0.70 (*t* < 0.35) for all nonsignificant comparisons, *n* = 14–16 for each diet × age combination; Table 1). Whiskers represent standard error. **(B)** Protein-fed flies had significantly higher LSP-2 protein abundance 3 through 9 days after adult eclosion (Two sample *T*-test, *indicates *p* < 0.05 (*t* > 2.85), **indicates *p* < 0.01 (*t* > 3.5), *p* > 0.1 (*t* < 1.75) for all nonsignificant comparisons, *n* = 14–16 for each diet × age combination; Table 1). Dashed black line indicates the age at which protein-fed males began calling significantly more than protein-deprived males. Whiskers represent standard error. **(C)** Representative Western blot showing that LSP-2 content was higher in protein-fed males.

Before we tested the extent to which protein storage affected male reproductive behavior, we first characterized and confirmed the identity of the major adult storage protein, LSP-2, in *A. suspensa* (Chrysanthis et al., 1994; Hahn et al., 2008; Supplementary Table 2; Supplementary Figures 1A,B). As expected, the abundance of *lsp-2* transcripts in whole body homogenates increased with age in protein-fed males, but decreased with age in protein-deprived males (LMM, Model B, Age^∗^Diet had an effect size of 0.242, S.E. of 0.0703, *p* < 0.01, *n* = 207, Table 1). Protein-fed males had significantly higher *lsp-2* transcript abundance than protein-deprived males 2–9 days after adult eclosion (Two sample *T*-test, for significant comparisons *p* < 0.05, *t* > 2.85, for all nonsignificant comparisons *p* > 0.70, *t* < 0.35, *n* = 14–16 for each diet × age combination; Figure 2A). Freshly eclosed males had low *lsp-2* transcript abundance, and both protein-fed and protein-deprived males had low *lsp-2* transcript abundance on the 1st day after adult eclosion. Protein-fed males increased their *lsp-2* transcript abundance over the first 4 days after adult eclosion, and *lsp-2* transcript abundance remained high 5–7 days after adult eclosion. In contrast, protein-deprived males retained low, almost undetectable *lsp-2* transcript abundance through 9 days of adulthood.

In protein-fed males, LSP-2 protein abundance in whole-body homogenates remained high until 7 days after eclosion, likely reflecting newly synthesized LSP-2 maintaining the high LSP-2 abundance carried over from larval life. In contrast, LSP-2 protein abundance fell dramatically 1 day after eclosion in protein-deprived males, likely due to the depletion of larvally derived LSP-2 (Figure 2B, western blot image in Figure 2C). LSP-2 protein abundance was significantly higher in protein-fed males than protein-deprived males starting 3 days after adult eclosion and throughout the rest of our sampling to 9 days after adult eclosion (Two sample *T*-test, *p* < 0.05 and *t* > 2.85 for all tests, *n* = 14–16 for each diet × age combination). *lsp-2* transcript abundance and age explained LSP-2 protein abundance (LMM, Model C, *lsp-2* transcript abundance had an effect size of 0.454, S.E. of 0.0903, *p* < 0.001, Age had an effect size of 0.383, S.E. of 0.0710, *p* < 0.001, *n* = 207). Males with higher *lsp-2* transcript abundance had significantly higher LSP-2 protein concentration (LMM, Model C, *lsp-2* transcript abundance had an effect size of 0.454, S.E. of 0.0903, *p* < 0.001, *n* = 207). Together, our results suggest that *lsp-2* expression is sensitive to dietary protein, leading to different LSP-2 protein titers circulating in the blood of protein-fed and protein-deprived males. These data are consistent with LSP-2 acting as an amino acid store in male *A. suspensa.*

### LSP-2 Abundance Between Dietary Treatments Diverges Before Behavior Diverges

If amino acid stores regulate reproductive displays, then LSP-2 protein abundance should diverge between protein-fed and protein-deprived males before their reproductive behavior diverges. We examined when LSP-2 abundance and sexual display behaviors diverged between protein-fed and protein-deprived males. LSP-2 abundance became significantly higher in protein-fed males than in protein-deprived males 1 day before the proportion of males exhibiting sexual display behavior significantly diverged between the two groups (Figure 2B). We examined the effect of age, diet, *lsp-2* transcript abundance, and LSP-2 protein abundance on sexual display behavior. In our fully parameterized model, LSP-2 protein abundance did not significantly influence sexual display behavior (GLMM, Model D, LSP-2 protein abundance had an effect size of 0.0340, S.E. of 0.124, *p* = 0.783, *n* = 207). But, because LSP-2 content and sexual displays both strongly covaried with time in each feeding regime (Pearson’s correlation, *r* = −0.17, *p* = 0.01, *n* = 207), we were unable to disentangle these effects and determine whether males with higher LSP-2 content called earlier, requiring a manipulative experiment.

### *lsp-2* Knockdown Mimics the Protein-Deprived Mating Phenotype

**FIGURE 3:**
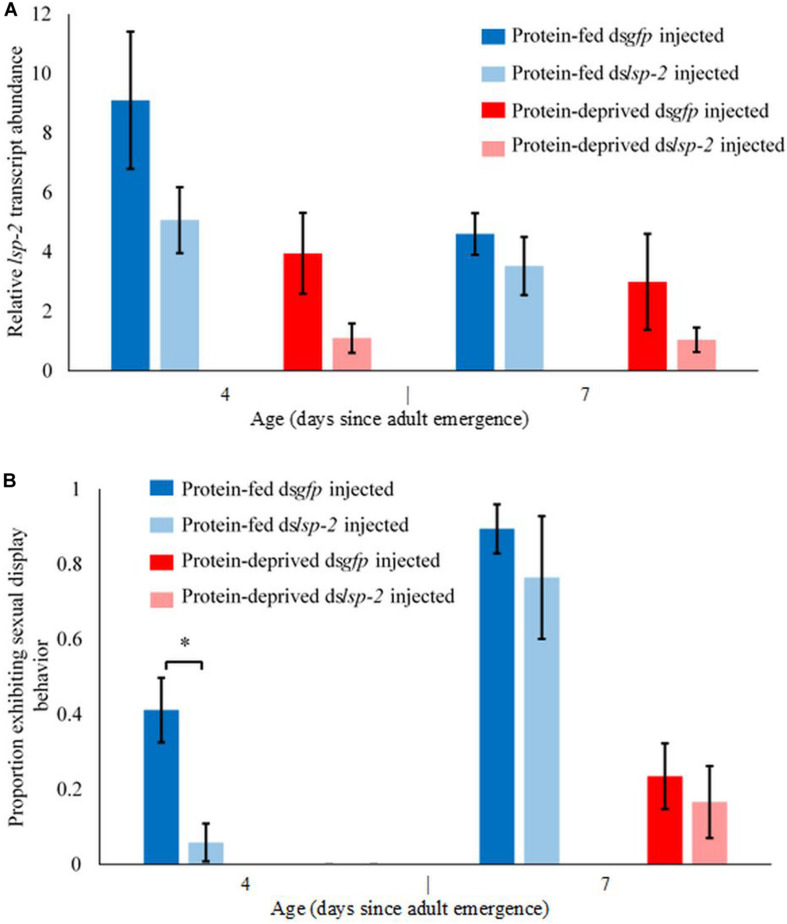
Anti-*lsp-2* dsRNA injection decreased *lsp-2* transcript abundance in whole animals and reduced the proportion of males exhibiting sexual display behavior. **(A)** Diet and injection treatment each independently significantly influenced *lsp-2* transcript abundance (GLMM (diet), *t* = 3.695, *p* < 0.001; GLMM (injection treatment), *t* = 2.75, *p* < 0.01, *n* = 123). Across all diets and ages, average *lsp-2* transcript abundance was ∼45% lower in anti*-lsp-2* dsRNA injected males compared to control dsRNA injected males. Whiskers represent standard error. **(B)** Protein-fed anti-*lsp-2* dsRNA injected males exhibit significantly less sexual display behavior than protein-deprived anti-*gfp* dsRNA injected males (Pearson’s Chi-squared, *χ*^2^ > 5.9, *p* = 0.0348, *n* = 27). However, the RNAi effect was no longer detectable 7 days after adult eclosion (RNAi treatment was absent from the reduced model, *n* = 70). As in previous experiments, protein fed males exhibited significantly more sexual display behavior than protein-deprived males (GLMM (diet), *z* = 4.64, *p* < 0.001, *n* = 12–18 for each diet × age × dsRNA treatment combination in both **(A,B)**. Whiskers represent standard error. Although *lsp-2* knockdown was incomplete and temporary, together our results suggest that LSP-2 regulates sexual display behavior in *A. suspensa*.

To test the extent to which LSP-2 abundance affects the timing and frequency of male mating behavior, we knocked down *lsp-2* transcript abundance in whole animals using RNAi. Average *lsp-2* transcript abundance was ∼45% lower in anti*-lsp-2* dsRNA injected males compared to control dsRNA injected males, suggesting incomplete but detectable knockdown (LMM, Model F, RNAi treatment had an effect size of 2.30, S.E. of 0.834, *p* < 0.01, *n* = 123; Figure 3A). However, the degree of knock down was much greater 4 days after adult eclosion than 7 days after adult eclosion (Figure 3A), likely due to a loss of RNAi efficacy with time since the flies were treated with dsRNA on the day of eclosion. Similar to our previous experiments, protein feeding increased *lsp-2* transcript abundance across all ages and RNAi treatments compared to protein-deprived anti*-lsp-2* dsRNA injected males and protein-deprived control dsRNA injected males (LMM, Model F, Diet had an effect size of 3.08, S.E. of 0.833, *p* < 0.001, *n* = 123; Figure 3A). However, *lsp-2* transcript abundance did not detectably change with age, though we only sampled from 2 ages and the age effect did trend toward significance (LMM, Model F, Age had an effect size of 1.76, S.E. of 0.863, *p* = 0.0721, *n* = 123; Figure 3A).

Protein-fed anti*-lsp-2* dsRNA injected males were significantly less likely to engage in sexual display behavior than protein-fed control dsRNA injected flies 4 days after adult eclosion (Pearson’s Chi-squared, *χ*^2^ = 4.5, *p* = 0.0348, *n* = 27, Figure 3B). Seven days after adult eclosion, significantly fewer protein-deprived males exhibited sexual display behavior than protein-fed males (GLMM, Model G, Diet had an effect size of 3.00, S.E. of 0.646, *p* < 0.001, *n* = 70). However, anti*-lsp-2* dsRNA injection did not significantly decrease sexual display behavior 7 days after adult eclosion in either the protein-deprived or protein-fed males (absent from reduced GLMM model, *n* = 70, Table 1G), perhaps due to incomplete knock down or loss of knock-down efficiency as time since *lsp-2* dsRNA injection increased. Altogether, our loss-of-function experiment nominates LSP-2 protein stores as a candidate regulatory mechanism for adult reproductive maturation and male sexual display behavior in a lekking fly.

## Discussion

### Protein Stored as LSP-2 Regulates Reproductive Displays

We show that protein stores regulate male tephritid behavioral reproductive maturation and sexual display behavior. Our conclusion is supported by four pieces of evidence. First, the hexamerin storage protein LSP-2 remained abundant in response to dietary protein availability in protein-fed flies, but was quickly depleted in protein-deprived flies. Second, protein-fed male flies began sexual display behavior earlier than protein-deprived males, and a greater proportion of protein-fed males engaged in sexual displays than protein-deprived males. Third, whole body LSP-2 abundance diverged between protein-fed and protein-deprived flies 1 day before their sexual display behavior diverges. Fourth, partial knockdown of *lsp-2* induced a detectable delay in male sexual display behaviors. Notably, we disentangle the effects of protein storage from the availability of dietary protein. We find that inability to store amino acids in LSP-2 mimics the effect of dietary protein-deprivation in *A. suspensa* males.

One caveat to our study is that the well-known difficulties of quantifying feeding in flies prevented us from directly measuring protein consumption. Could the delay in the onset of male mating displays we observed in our *lsp-2* RNAi treatment relative to *gfp* RNAi control flies have been caused by *lsp-2* RNAi males eating less than *gfp* RNAi control flies? To determine whether our *lsp-2* RNAi treated males may have consumed less protein than *gfp* RNAi controls, we estimated total body soluble protein in both treatment groups. We detected no difference in soluble protein content between *lsp-2* RNAi injected males and *gfp* RNAi control males. Yet, we were able to detect that protein-deficient males had lower soluble protein content than protein-fed males. Thus, although we did not quantify protein feeding directly, we believe that the protein-fed *lsp-2* RNAi males were not generally protein malnourished, suggesting their lack of sexual display behavior was due to lack of LSP-2 protein stores specifically rather than general protein deficiency. We did not investigate the fate of ingested amino acids in *lsp-2* RNAi males for this study, though we suspect ingested amino acids were still being used as anabolic substrate for the growth of secondary sexual organs and production of sperm. Our study illustrates that males, like females, regulate their reproductive behavior based on their nutrient stores (Houston et al., 2006; Soulsbury, 2019). We also found that often overlooked amino acid capital can regulate breeding in an insect. Insects are a highly abundant and diverse class of animals many of which have complex and costly mating behaviors, so the role of amino acid capital and hexamerins in mating behavior warrant further study.

### Hexamerins May Signal Protein Stores

Capital-breeding is an important reproductive strategy that allows animals to store nutrients when they are abundant and then use nutrient stores later to fuel reproduction. Many animals rely on some combination of nutrient capital and income, and can regulate their behavior based on whether they have adequate nutrient stores to support reproduction (Frisch, 1985; Yin et al., 1999; Teal et al., 2013; Lebreton et al., 2017). The connection between fat storage and female fertility is well known, but our understanding of the relationships between male reproductive behavior and stored nutrition is incomplete (Mirth and Piper, 2017; Soulsbury, 2019). Although fat stores and leptin are reported to promote puberty in vertebrate males, the evidence from humans and rodents is still mixed and excessive fat stores may even inhibit reproduction (Zhang and Gong, 2018). Other studies have found that dietary protein availability regulates reproductive behavior in tephritid male flies, but none have explicitly tested the role of protein stores (Warburg and Yuval, 1997; Marchini et al., 2003; Teal et al., 2013). Our study addresses this gap, finding that protein stores regulate reproductive behavior in male tephritids.

We found that the hexamerin storage protein LSP-2 modulates sexual display behavior in male *A. suspensa*, suggesting that hexamerin storage proteins could be one regulator of reproduction in insects. Hexamerin storage proteins are an arthropod-specific family of proteins that diverged from arthropod hemocyanins early in insect evolution (Burmester, 1999). Hexamerin storage proteins have been found in every insect species that has been investigated for their presence (Burmester, 1999). Hexamerins are also present in the closely related hexapod, Diplura (Xie and Luan, 2014), and even have close homologs in crustaceans (Burmester, 1999; Terwilliger et al., 1999). Hexamerins accumulate prior to anabolically demanding life history transitions in many insects, including metamorphosis, diapause, and female reproduction (Pan and Telfer, 1996; Wheeler and Buck, 1996; Burmester, 1999; Hahn and Denlinger, 2007). However, the ability of hexamerin storage proteins to regulate life history transitions has only been cursorily tested, partially because complete hexamerin knockdown is difficult (Tokar et al., 2014; Li et al., 2017). Because many insects have multiple hexamerin storage proteins, knockdown of one hexamerin may induce functional compensation by overexpression of another hexamerin storage protein (Tokar et al., 2014; Li et al., 2017). Higher flies (Brachycera) like *A. suspensa* and *D. melanogaster* also have multiple hexamerins expressed during larval life, but only *lsp-2* is expressed during the adult stage (Chrysanthis et al., 1994; Capurro et al., 2000; Hahn et al., 2008). Thus, knocking down *lsp-2* provides an opportunity to examine the functional roles of hexamerins. However, even in our study knockdown of *lsp-2* appears temporary. We injected dsRNA on the day of adult eclosion, and knockdown efficiency was much greater 4 days after injection compared to 7 days after injection. Future studies in other insects should perform knockdown or overexpression of hexamerins individually and in combination to investigate their roles in the timing of life history events. Such experiments could also test the extent to which regulatory roles of hexamerin storage proteins are general across insects. Future studies could also use diet switching to test the relative importance of short- and long-term dietary protein availability, protein feeding, and protein storage in regulating behavior. However, our finding that *lsp-2* knockdown suppressed reproductive behavior suggests that protein stores can indeed regulate male reproduction.

For any animal to use their nutrient stores to regulate their reproductive behavior, as we observed with protein stores in *A. suspensa*, peripheral tissues must communicate a measure of their stores with the brain. One mechanism that animals use to measure and communicate their nutrient stores is circulating signals, hormones. For example, tetrapods secrete a peptide hormone, leptin, that measures stored fat and coordinates this information with growth, development, metabolism (Woods et al., 1998; Mantzoros, 2000; Paolucci et al., 2001; Londraville et al., 2017). Our understanding of leptin function is best developed in mammals: mammalian adipose tissue secretes leptin into the blood and leptin is sensed by receptors in the secretory cells of the brain and pancreas (Woods et al., 1998). The leptin signal coordinates feeding, growth, metabolism, and reproduction with fat stores (Woods et al., 1998; Mantzoros, 2000). How flies and other insects sense their nutrient stores is less clear. In *D. melanogaster*, a leptin-like hormone, unpaired 2, is secreted into the blood when the fly consumes dietary fat (Rajan and Perrimon, 2012; Londraville et al., 2017). However, whether unpaired 2 is sensitive to fat stores is unclear. Even more unclear is the mechanism (s) for sensing protein stores in insects. We propose that insects use the titers of hexamerin storage proteins like *lsp-*2 as a circulating signal. In support of this hypothesis, our findings suggest that (i) LSP-2 circulates in the blood of *A. suspensa*, (ii) LSP-2 levels providing a reliable signal of protein store quantity, (iii) *lsp-2* knockdown mimics dietary protein deprivation, and (iv) LSP-2 is secreted by an important nutrient-signaling tissue in insects, the fat body. More broadly, we suggest that the primary role of hexamerins is protein storage, but that specific hexamerins may also have dual roles in storage and as a circulating signal secreted by the fat body. Any circulating signal must also have a receptor, and one receptor of hexamerins has been identified, *fat body protein 1 (fbp-1)* (Burmester and Scheller, 1999). FBP-1 has previously been shown to participate in receptor-mediated uptake of hexamerins by the fat body immediately before metamorphosis in holometabolous larvae (Burmester and Scheller, 1999). Interestingly, transcripts for *fbp-1* have also been detected in single-cell transcriptomes of *D. melanogaster* brain neurons (Davie et al., 2018), though FBP-1 specific protein detection is still needed. In *D. melanogaster, fbp-1* transcripts are also still found in the in the adult fat body (Kadener et al., 2006). We propose that FBP-1 in the fat body binds LSP-2 to liberate the amino acids for anabolic functions, while FBP-1 in the brain binds LSP-2 to provide a measure of condition and transduce this information to modulate behavior. Our hypotheses are consistent with both *in vivo* and *ex vivo* studies indicating that the insect fat body secretes one or more nutritional hormones, termed fat-body-derived signals, that communicate amino acid status to the brain and reproductive tissues in *D. melanogaster* (Géminard et al., 2009; Sousa-Nunes et al., 2011). In response to fat-body-derived signals, the brain and reproductive tissues accelerate growth and reproductive development (Géminard et al., 2009; Armstrong et al., 2014). However, the number and identity of fat-body-derived signals remains unclear. Furthermore, the role of fat-body signals in sensing short-term amino acid income and stored amino acid reserves is also unclear. In *D. melanogaster*, fat body derived signals generate brain and peripheral tissue responses distinct from those generated by circulating amino acids (Géminard et al., 2009; Armstrong et al., 2014). Colombani et al. (2003) and Arquier et al. (2008) propose that acid-labile protein subunit is a fat-body-derived signal that forms a complex with insulin-like peptides to coordinate amino acid status with growth. Similarly, Koyama and Mirth (2016) propose that growth-blocking peptides are fat-body-derived signals that stimulate insulin-like peptide release from the brain. These models both account for currently circulating amino acids, but not the longer-term amino acid reserves in stored protein.

No fat-body-derived signal has been proposed that communicates stored protein, yet protein stores are a critical part of insect life-histories from molting to reproduction (Burmester, 1999). We hypothesize that hexamerins like LSP-2 may act as fat-body-derived signals that indicate protein stores directly. Our results are consistent with a signaling role for LSP-2, but do not provide sufficient evidence to fully support our hypothesis. Consistent with our hypothesis, hexamerins can act as mitogens inducing cell proliferation in the midgut of molting lepidopterans and the reproductive organs of honeybees (Blackburn et al., 2004; Hakim et al., 2007; Martins et al., 2011). Hexamerins supply amino acids during molting and reproduction (Pan and Telfer, 1996; Burmester, 1999; Arrese and Soulages, 2010), so hexamerin abundance may signal that molting and reproduction can proceed because the requisite amino acids have been stored. Hexamerins also have been implicated in caste differentiation in termite colonies and *Polistes* wasp colonies (Zhou et al., 2006; Hunt et al., 2007). Nutrition controls caste differentiation in termites and wasps (Scharf et al., 2007; Berens et al., 2015), so hexamerin accumulation could act as a link between nutrient intake and caste differentiation. Juvenile hormone (JH) is also implicated in caste differentiation, and both Braun and Wyatt (1996) and Zhou et al. (2006) have proposed that hexamerins play a functional role modifying juvenile hormone (JH) signaling. In addition to well-known roles regulating juvenile development, JH is also known to regulate reproduction in most insects (Riddiford, 2012). In adult male flies, including *D. melanogaster*, *A. suspensa*, and many other tephritids, application of methoprene (a JH analog) increases male courtship behavior (Teal et al., 2013; Wijesekera et al., 2016). In many tephritids, dietary protein during adulthood and methoprene treatment have an additive effect in promoting sexual display behavior (Teal et al., 2013), suggesting that high LSP-2 titers induced by protein feeding may increase the potency of JH signaling. Further research is needed to clarify whether hexamerins and JH may have additive or synergistic effects in inducing sexual display behavior in *A. suspensa*. More work is also needed to test whether hexamerin storage proteins generally perform a regulatory role during nutrient intensive insect life-history transitions like caste differentiation and reproduction.

Understanding reproduction in male insects also has substantial practical application because many pest insects, especially tephritid fruit flies, are controlled by the sterile insect technique wherein lab-grown sterile males are released into the field to compete with wild males. Sterile male release is an environmentally friendly alternative to chemical insecticides, but is often more expensive than chemical alternatives (Bakri et al., 2005). Dietary protein, methoprene, and plant volatiles are often used in sterile insect technique programs to promote reproductive behaviors in sterile males (Teal et al., 2013; Segura et al., 2018). The success of sterile insect technique programs is predicated on the ability of sterile males to exhibit reproductive displays accurately enough and frequently enough to compete with wild males. Our findings suggest artificially upregulating the LSP-2 signal could potentially accelerate and increase reproductive behavior of tephritids. Hyper-sexual males could improve the efficacy of sterile male biological control agents and decrease the cost of environmentally friendly pest control programs that release sterile males.

## Data Availability Statement

The R code used to analyze data and datasets themselves have been made available in the Supplementary Material.

## Author Contributions

CS carried out the molecular lab work and behavioral assays, collected and analyzed data, participated in the design of the study, and drafted and revised the manuscript. JH participated in conceiving of the study and critically revised the manuscript. DH participated in conceiving and designing the study and helped draft and revise the manuscript. All authors contributed to the article and approved the submitted version.

## Conflict of Interest

The authors declare that the research was conducted in the absence of any commercial or financial relationships that could be construed as a potential conflict of interest.
