# Supplementary material for: Protein Stores Regulate When Reproductive Displays Begin in the Male Caribbean Fruit Fly
Source: Front Physiol. 2020 Aug 6;11:991. doi: 10.3389/fphys.2020.00991 (PMC7424033; doi:10.3389/fphys.2020.00991)
Supplement: Supplementary file 1 [file Presentation_1.pdf]

Supplementary Information

SI Table 1. Primer sequences for degenerate primers, *lsp-2*, and *rp-18* and RNAi.

Oligonucleotides	Sequence (5’→3’)
lsp-2 degenerate primer FWD	TCATCCACGAACACAAG
lsp-2 degenerate primer REV	CTATTGGCATATGGCTGAGG
A. suspensa lsp-2 FWD	ACCATTGAGTTGGCTGAGAGGTATAG
A. suspensa lsp-2 REV	AGGAAGTATGCGAGCTGGTGAT
A. obliqua rp18 FWD (Nakamura et al., 2016)	ACCATTGAGTTGGCTGAGAGGTATAG
A. obliqua rp18 REV (Nakamura et al., 2016)	AGGAAGTATGCGAGCTGGTGAT
Anti-*lsp-2* FWD	TAATACGACTCACTATAGGGGTTGGCTGAGAGGTATAGAATG
Anti-*lsp-2* REV	TAATACGACTCACTATAGGGCTCCATATCGTCCTTGTGTTC
gfp FWD	TAATACGACTCACTATAGGGGTGATGTTAATGGGCACAAAT
gfp REV	TAATACGACTCACTATAGGGTGTTGATAATGGTCTGCTAGTT

SI Table 2. BLAST results for our putative *A. suspensa* LSP-2 amplicon.

Alignment	Total Score	Query cover	E value	Identity	Accession
PREDICTED: *B. oleae* LSP-2 (LOC106624631), mRNA	625	96%	4e-176	82%	XM_014244390.1
PREDICTED: *B. cucurbitae* LSP-2 (LOC105213296), mRNA	608	93%	4e-171	82%	XM_011186009.1
PREDICTED: *B. dorsalis* LSP-2 (LOC105225725), mRNA	525	96%	4e-146	80%	XM_011204324.2
PREDICTED: *C. capitata* LSP-2 (LOC101449410), mRNA	540	99%	2e-150	80%	XM_004530624.3
D. melanogaster LSP-2 gene	221	60%	1e-56	71%	X97770.1

SI Table 3. Sequence of dsRNAs used in RNAi experiments

dsRNA identity	Sequence
Anti-*lsp-2*	GTTGGCTGAGAGGTATAGAATGCACAATTTCCACCAGGTAAA<br>GAAGTTGGACAACGTTTACAATGTTGCTATCAAGGCGAACTA<br>CACCAACGTATACGGCAACCTACATGGCGATCACCAGCTCGC<br>ATACTTCCTCGAGGATGTTGGTCTTAATTCCTTCTACTACTAC<br>TACAACTTAGATTATCCGTACTGGACTAAGGGTGTCGAAGGT<br>TATGAGTTAAACAAGGATCGTCGTGGTGAGTTCTGGATCTAC<br>ACGCATTGGCAGTTGTTGGCTCGCTACTATCTGGAGCGTTTGT<br>CTCACGGTTTGGGTGAAATTGAGGACTTTGACATGTACGAGY<br>CTGTTGTCAATGGCTACCACAGTGGCTTGCGTTACTATCCTGG<br>TGTGAGTTACCCCAACCGTGACAATGGCTACAGCTTTTACCAT<br>ATCGAGAATATGGAGCACATGCGCATGATTCATTTAATTAGC<br>GTACGCATTATGAATTTCATTCACGGTGAACACAAGGACG
Anti-*gfp*	TGATGTTAATGGGCACAAATTTTCTGTCAGTGGAGAGGGTGAA<br>GGTGATGCTACATACGGAAAGCTTACCCTTAAATTTATTTGCAC<br>TACTGGAAAACTACCTGTTCCATGGCCAACACTTGTCACTACTT<br>TCTCTTATGGTGTTCAATGCTTTTCCCGTTATCCGGATCATATGA<br>AACGGCATGACTTTTTCAAGAGTGCCATGCCCGAAGGTTATGTA<br>CAGGAACGCACTATATCTTTCAAAGATGACGGGAACTACAAGA<br>CGCGTGCTGAAGTCAAGTTTGAAGGTGATACCCTTGTTAATCGT<br>ATCGAGTTAAAAGGTATTGATTTTAAAGAAGATGGAAACATTC<br>TCGGACACAAACTCGAGTACAACTATAACTCACACAATGTATA<br>CATCACGGCAGACAAACAAAAGAATGGAATCAAAGCTAACTT<br>CAAAATTCGCCACAACATTGAAGATGGATCCGTTCAACTAGCA<br>GACCATTATCAACA

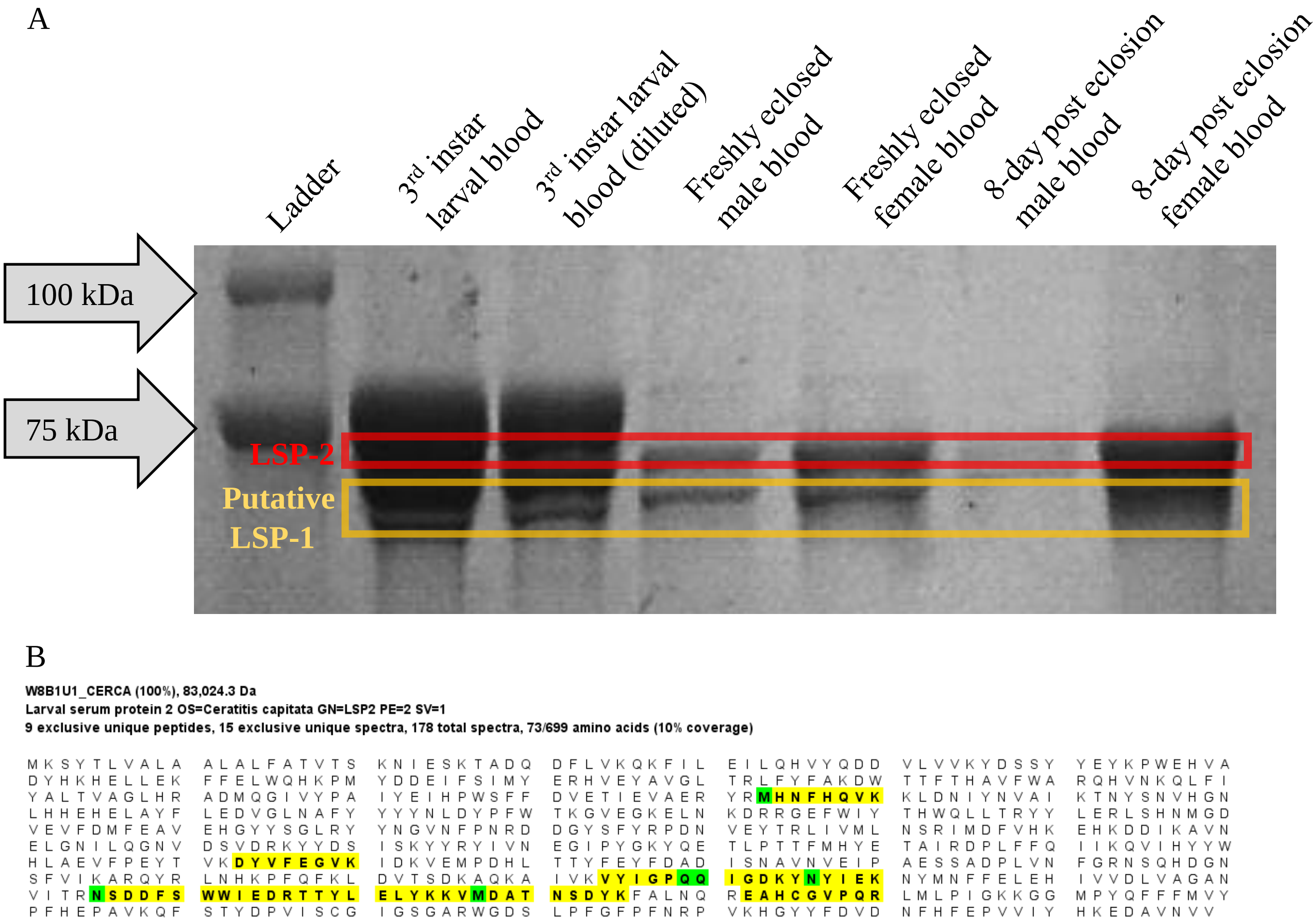

SI Figure 1. Characterization of LSP-2 in *A. suspensa*. (A) Protein in the blood of *A. suspensa*, separated by SDS-PAGE and stained with Coomassie biosafe (Bio-Rad, Hercules, CA). The bands are, from left to right; Ladder, blood from 3rd third instar larvae, blood from wandering 3rd instar larvae that is diluted to more clearly show protein bands, freshly eclosed male blood, freshly eclosed female blood, blood from males 8 days after adult eclosion, and blood from females 8 days after adult eclosion. The red box encompasses LSP-2 protein bands, as confirmed by LC/MS-MS, and the yellow box encompasses putative LSP-1 bands (3 similar hexamerins that are not expressed during adulthood; Beneš et al., 1990) that were not tested in this study. LSP-2 persists into the 8th day of adult life, but putative LSP-1 bands are depleted by this age. (B) Results of LC-MS/MS of the LSP-2 band. Protein extracted from band that was excised from

an SDS-PAGE gel containing protein from a protein-fed male *A. suspensa* 8 days after adult eclosion. Nine unique peptide fragments (in yellow) were identified that match the reference sequence: predicted LSP-2 sequence of *C. capitata*. Modified amino acids are highlighted green. 10% of the reference protein sequence was covered by our LC-MS/MS fragments. Together, these results strongly support that the band we have focused on contains LSP-2 protein in *A. suspensa*.

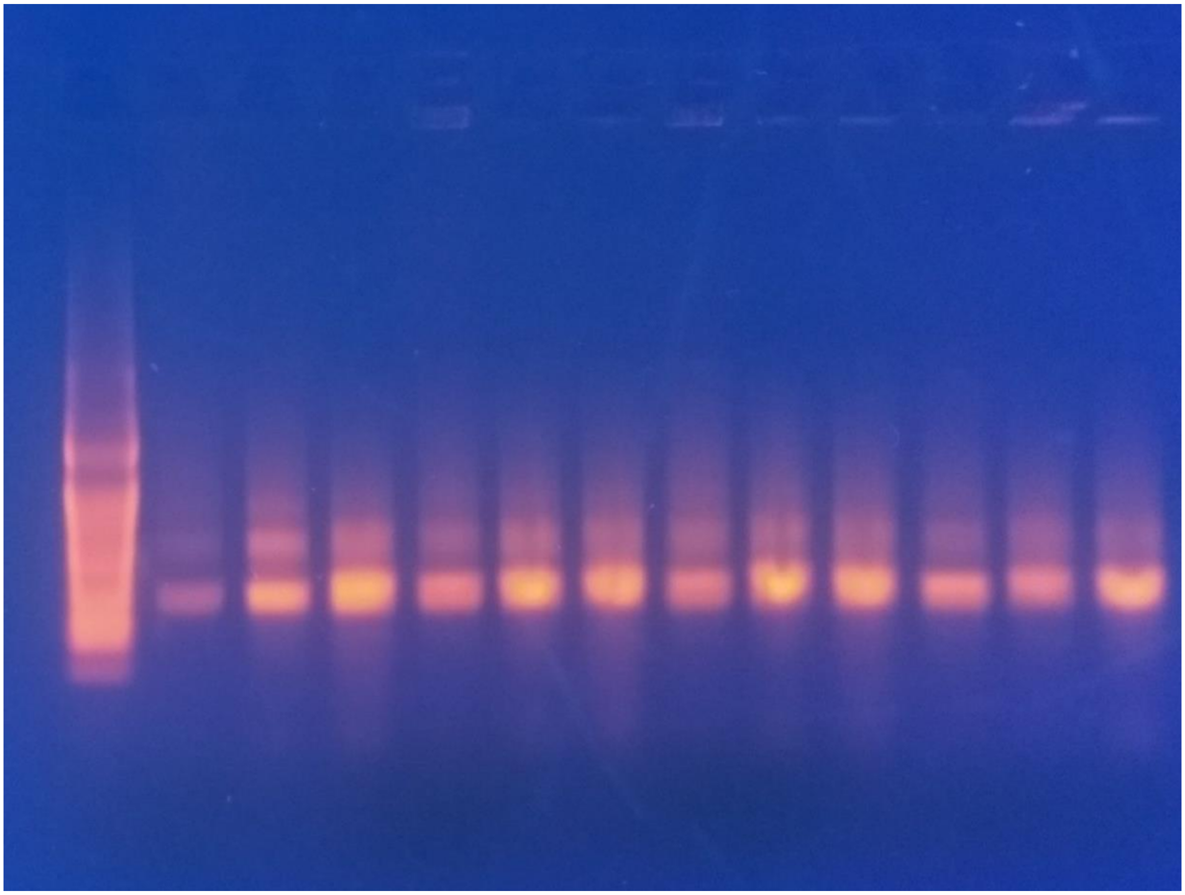

SI Figure 2. Bleach gel electrophoresis to check RNA quality. Every 24th RNA isolation was selected for quality assessment. Lane 1 is an RNA ladder and lanes 2-13 are RNA samples. 5 µg

RNA was loaded into each well. Upper and lower bands correspond to large and small rRNA subunits that are visualized with ethidium bromide excited by UV light.

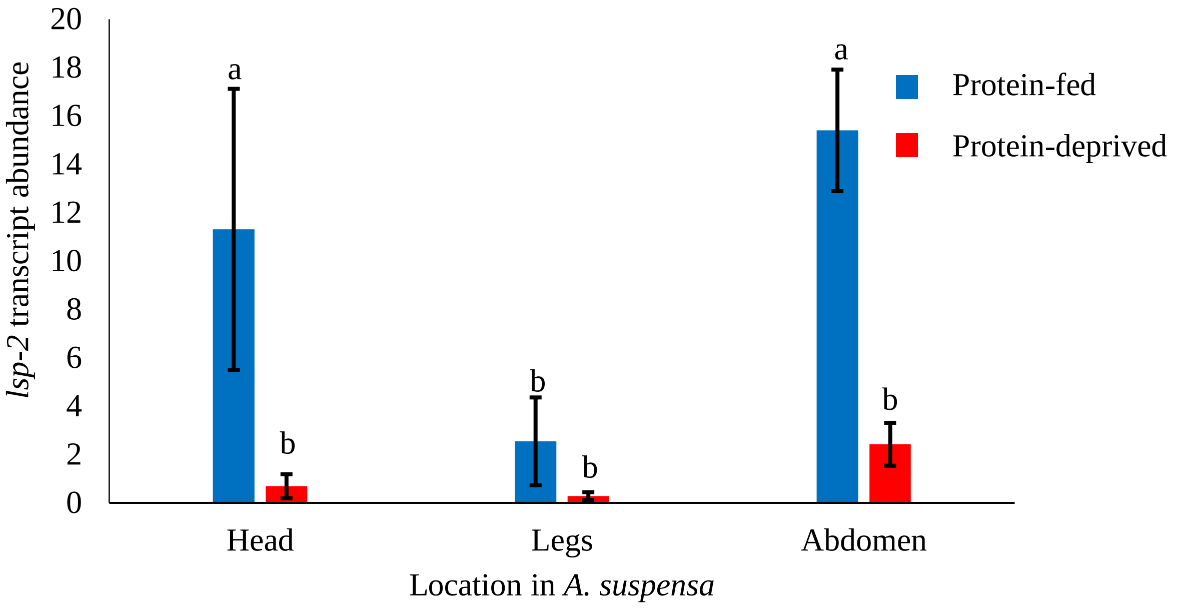

SI Figure 3. Abundance of *lsp-2* transcripts in protein-fed and protein-deprived flies 4 days after adult eclosion. Bars represent the average transcript abundance of 4 samples consisting of pooled tissue from 5 individuals. Blue bars indicate abundance in tissue from protein-fed flies, while red bars indicate abundance in tissue from protein-deprived flies. Error bars represent S.E. *lsp-2* transcript abundance was significantly elevated in the head and abdomen of protein-fed flies (Tukey's post-hoc test, $p<0.05$ for both tests, n=21), corresponding to the location of fat body in the adult fly.

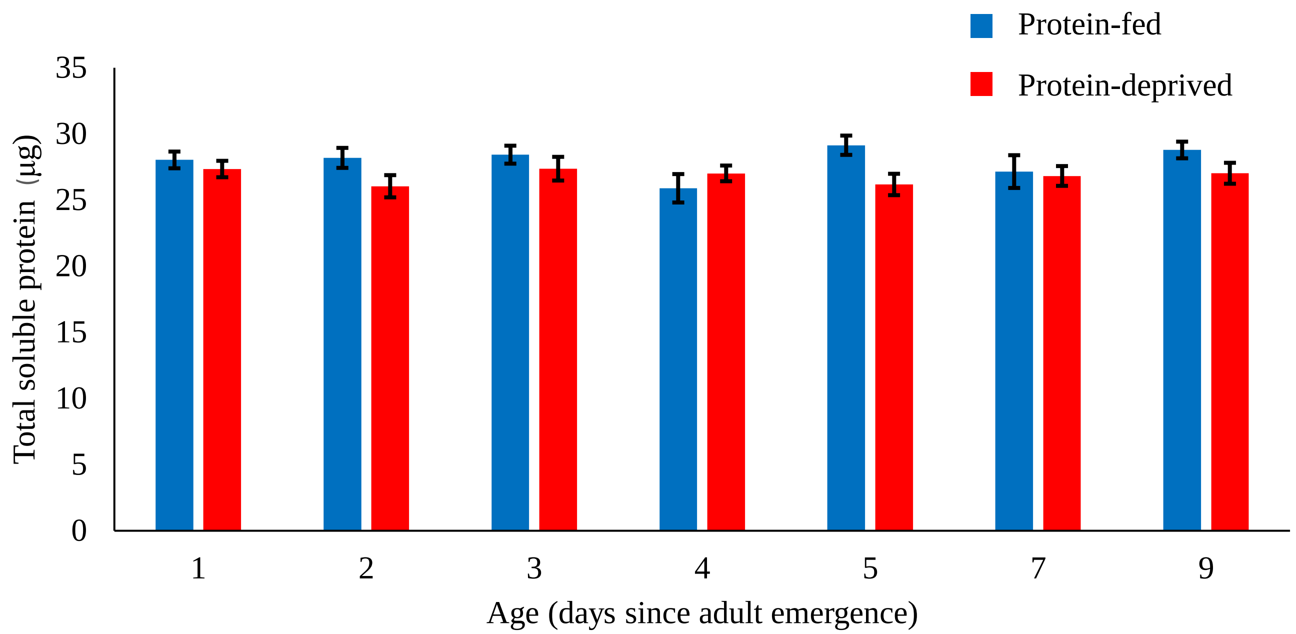

SI Figure 4. Total soluble protein content from whole bodies of protein-fed and protein-deprived flies 1 to 9 days after adult eclosion. Blue bars represent the total soluble protein content of protein-fed flies, while red bars represent the total soluble protein content of protein-deprived flies. Whiskers represent standard error. In a mixed linear model examining this data, protein-fed males had significantly higher total soluble protein content than protein-deprived males (LMM, Total protein ~ Diet, cohort as random factor, Diet had an effect size of 1.06, S.E. of 0.431, $p = 0.014$, $n = 207$). In post-hoc analysis, protein-fed flies did not have significantly higher total soluble protein content on any specific day (Two sample T-test, $p > 0.07$ and $t < 2.73$ for all tests, n = 14-16 for each diet x age combination).

SI Sequence 1. Partial mRNA sequence of LSP-2 in *Anastrepha suspensa*, as obtained via Sanger Sequencing. 5' to 3' orientation.

TCNTGCCGTC ATCTGGGCTC GTATGCATGT CAACAAACAG TTGTTCATAT

ACGCGCTGAC TGTGGCCGGT CTGCACCATG CCGATATGCA AGGCATTGTC

TACCCAGCCA TCTACGAAAT TCACCCATGG AGCTTCTTCG ATGTGGACAC

CATTGAGTTG GCTGAGAGGT ATAGAATGCA CAATTTCCAC CAGGTAAAGA

AGTTGGACAA CGTTTACAAT GTTGCTATCA AGGCGAACTA CACCAACGTA

TACGGCAACC TACATGGCGA TCACCAGCTC GCATACTTCC TCGAGGATGT

TGGTCTTAAT TCCTTCTACT ACTACTACAA CTTAGATTAT CCGTACTGGA

CTAAGGGTGT CGAAGGTTAT GAGTTAAACA AGGATCGTCG TGGTGAGTTC

TGGATCTACA CGCATTGGCA GTTGTTGGCT CGCTACTATC TGGAGCGTTT

GTCTCACGGT TTGGGTGAAA TTGAGGACTT TGACATGTAC GAGTCTGTTG

TCAATGGCTA CCACAGTGGC TTGCGTTACT ATCCTGGTGT GAGTTACCCC

AACCGTGACA ATGGCTACAG CTTTTACCAT ANCGAGAATA TGGAGCACAT

GCGCATGATT CATTTAATTA CCGTACGCAT TATGAATTTC ATTCACGGTG

AACACAAGGA CGATATGGAG GCTGTCAACC AGTTGGGCAA CATCTTACAA

GGCAATGTTG ATAGTGTTGA CAGAAAATTC TATAACAGCA TCAGCAAGAT

SI Sequence 2. Predicted protein sequence from the partial mRNA sequence of LSP-2 in *Anastrepha suspensa*. N-terminus to C-terminus orientation.

AVIWARMHVNKQLFIYALTVAGLHHADMQGIVYPAIYEIHPWSFFDVDTIELAERYRM
HNFHQVKKLDNVYNVAIKANYTNVYGNLHGDHQLAYFLEDVGLNSFYYYYNLDYPY
WTKGVEGYELNKDRRGEFWIYTHWQLLARYYLERLSHGLGEIEDFDMYESVVNGYHS
GLRYYPGVSYPNRDNGYSFYHXENMEHMRMIHLITVRIMNFIHGEHKDDMEAVNQLG
NILQGNVDSVDRKFYNSISK
